# Supra­molecular hydrogen-bonding patterns in the N(9)—H protonated and N(7)—H tautomeric form of an *N^6^*-benzoyl­adenine salt: *N* ^6^-benzoyl­adeninium nitrate

**DOI:** 10.1107/S2056989015024871

**Published:** 2016-01-09

**Authors:** Ammasai Karthikeyan, Nithianantham Jeeva Jasmine, Packianathan Thomas Muthiah, Franc Perdih

**Affiliations:** a School of Chemistry, Bharathidasan University, Tiruchirappalli 620024, Tamilnadu, India; b Faculty of Chemistry and Chemical Technology, University of Ljubljana, Vecna pot 113, PO Box 537, SI-1000 Ljubljana, Slovenia

**Keywords:** crystal structure, *N^6^*-benzoyl adenine, nitrate anion, hydrogen bonding, supra­molecular architecture

## Abstract

In the title molecular salt, C_12_H_10_N_5_O^+^·NO_3_^−^, the adenine unit has an *N* ^9^-protonated N(7)—H tautomeric form with non-protonated N^1^ and N^3^ atoms. The dihedral angle between the adenine ring system and the phenyl ring is 51.10 (10)°. The typical intra­molecular N^7^—H⋯O hydrogen bond with an *S*(7) graph-set motif is also present. The benzoyl­adeninium cations also form base pairs through N—H⋯O and C—H⋯N hydrogen bonds involving the Watson–Crick face of the adenine ring and the C and O atoms of the benzoyl ring of an adjacent cation, forming a supra­molecular ribbon with *R*_2_^2^(9) rings. Benzoyl­adeninum cations are also bridged by one of the oxygen atoms of the nitrate anion, which acts as a double acceptor, forming a pair of N—H⋯O hydrogen bonds to generate a second ribbon motif. These ribbons together with π–π stacking inter­actions between the phenyl ring and the five- and six-membered adenine rings of adjacent mol­ecules generate a three-dimensional supra­molecular architecture.

## Abstract 2

The N^9^—H protonated and N^7^—H tautomeric form of *N* ^6^-benzoyl­adenine cations are bridged by one of the oxygen atoms of the nitrate anion *via* N—H⋯O hydrogen bonds, generating a ribbon motif. The cations also form base pairs *via* N—H⋯O and C—H⋯N hydrogen bonds.

## Chemical context   

Non-covalent inter­actions, such as hydrogen bonding, halogen bonding and π–π inter­actions play major roles in mol­ecular recognition and pharmaceutical drug design processes (Desiraju, 1989 ▸; Perumalla & Sun, 2014 ▸). *N* ^6^-substituted adenine compounds continue to attract inter­est due to their biological activity as they can act as plant hormones and have anti-allergenic, anti­bacterial, anti­viral and anti­fungal properties (Hall, 1973 ▸; McHugh & Erxleben, 2011 ▸). *N* ^6^-substituted adenine compounds also exhibit an extensive variety of hydrogen-bonding patterns and supra­molecular architectures (Raghunathan & Pattabhi, 1981 ▸; Nirmalram *et al.*, 2011 ▸; Tamilselvi & Mu­thiah, 2011 ▸; McHugh & Erxleben, 2011 ▸; Jennifer *et al.*, 2014 ▸). The present investigation deals with the nitrate salt of *N* ^9^-protonated benzoyl­adenine (I). Nitrate ions are known to play pivotal roles in hydrogen bonded supra­molecular architectures, as they have three oxygen atoms to act as good hydrogen bond acceptors (Murugesan *et al.*, 1997 ▸; Cherouana *et al.*, 2003 ▸; Balasubramani *et al.*, 2005 ▸; Nirmalram *et al.*, 2011 ▸).

## Structural commentary   

**Figure 1:**
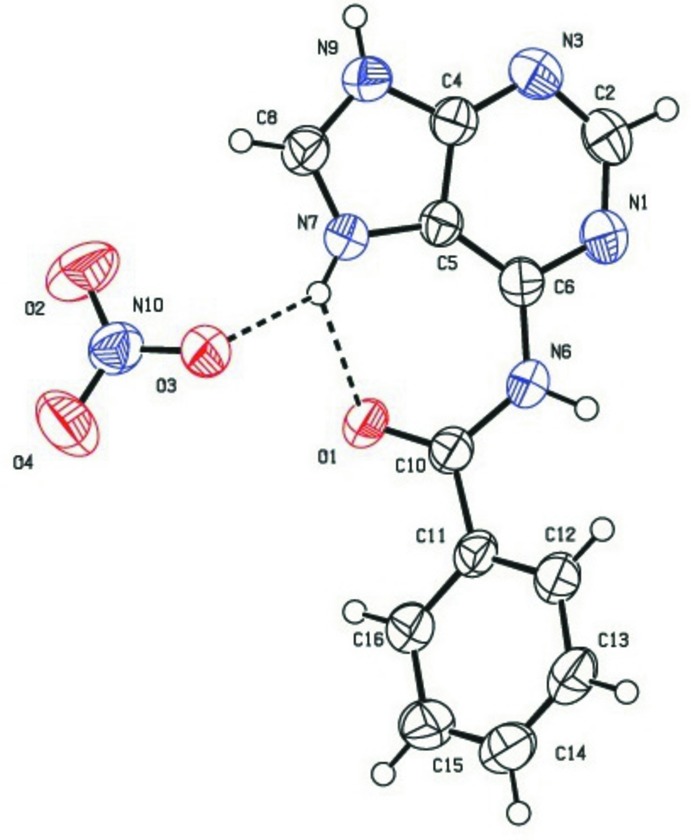
The asymmetric unit of the title compound showing the atom-numbering scheme. Displacement ellipsoids are drawn at the 50% probability level. Dashed lines represent hydrogen bonds.

The asymmetric unit of compound (I) consists of one *N* ^6^-benzoyl­adeninium cation and one nitrate anion, Fig. 1 ▸. In this salt, the *N* ^6^-benzoyl­adenine moiety is found in the N(7)—H tautomeric form with N9 protonated and N1, N3 non-proton­ated. The inter­nal angles at N7 [C8—N7—C5 = 108.9 (2)°] and N9 [C8—N9—C4 = 107.9 (2)°] are similar as both carry hydrogen atoms (Raghunathan & Pattabhi, 1981 ▸; Raghunathan *et al.*, 1983 ▸; Nirmalram *et al.*, 2011 ▸; Tamilselvi & Mu­thiah, 2011 ▸; García-Terán *et al.*, 2004 ▸; Bo *et al.*, 2006 ▸). The inter­nal angles at N1 [C6—N1—C2 = 118.9 (3)°] and N3 [C4—N3—C2 = 111.0 (3)°] agree with those reported for the neutral six-membered rings in other ademine structures (Raghunathan & Pattabhi, 1981 ▸; Karthikeyan *et al.*, 2015 ▸). An intra­molecular N7—H7⋯O1 hydrogen bond (Table 1 ▸) is observed on the Hoogsteen face of the purine ring with the benzoyl oxygen atom, generating an *S*(7) ring motif. A similar bond was found in the crystal structure of the neutral *N* ^6^-benzoyl adenine (Raghunathan & Pattabhi, 1981 ▸). The dihedral angle between the adenine ring system and the phenyl ring is 51.10 (10)°, and the C6—N6—C10—C11 torsion angle is −168.8 (2). The bond lengths and bond angles for the nitrate anion are in good agreement with literature values (Nirmalram *et al.*, 2011 ▸). Tables comparing dihedral and torsion angles in the title compound with those in related structures appear in the supporting information.
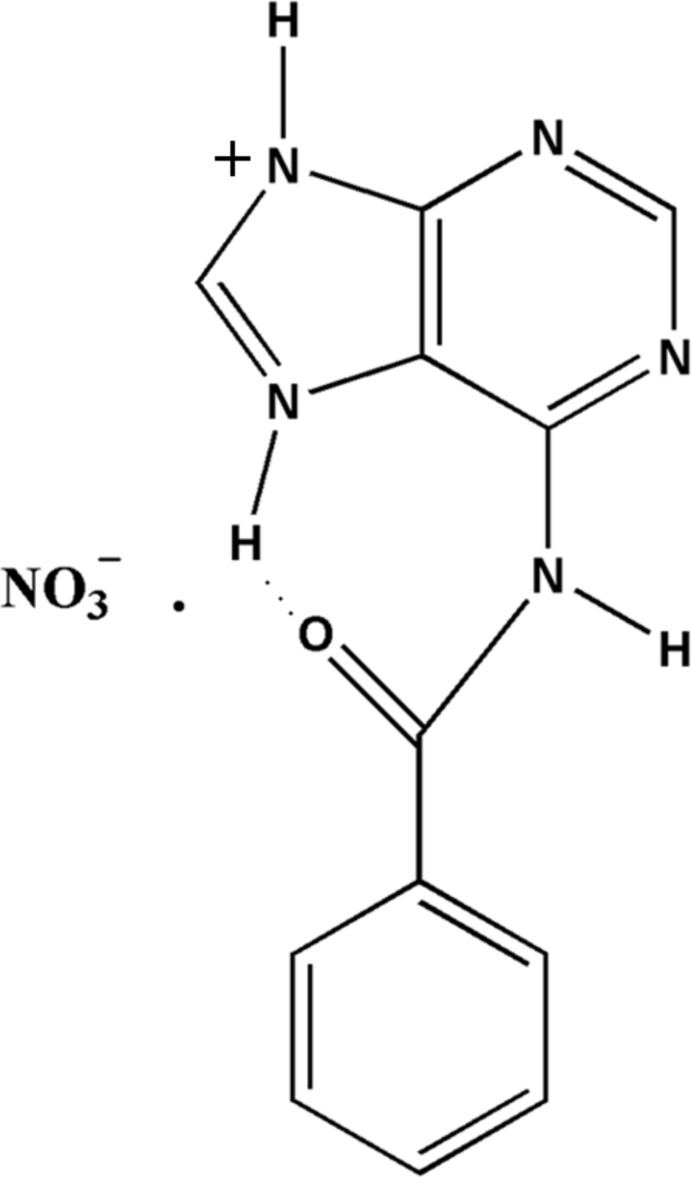


## Supra­molecular features   

**Figure 2:**
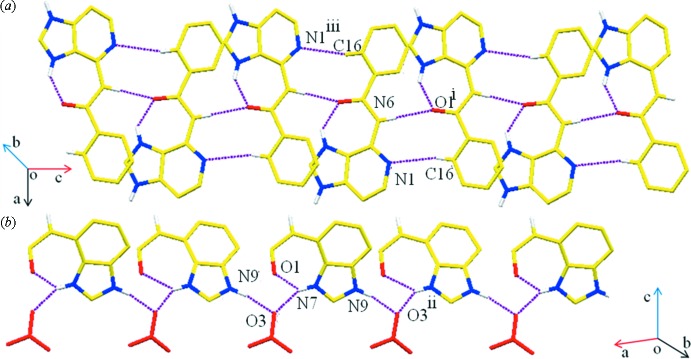
A view of two supra­molecular ribbons of (I). (*a*) A view of adeninium–benzoyl inter­actions *via* N—H⋯O and C—H⋯N hydrogen bonding, forming a supra­molecular ribbon. (*b*) A view of adeninum cations bridged by one of the oxygen atoms of the nitrate anion *via* N9—H9⋯O3 and N7—H7⋯O3 hydrogen bonds (purple dashed lines), generating a second type of ribbon motif. The phenyl groups and H atoms not involved in hydrogen bonding have been omitted for clarity. The symmetry codes are as given in Table 1 ▸.

**Figure 3:**
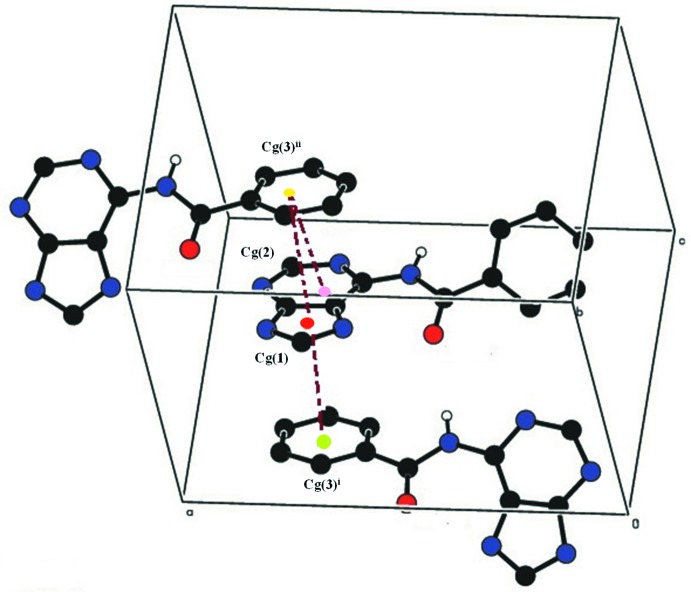
A view of π–π stacking inter­actions in (I). *Cg*1 is the centroid of the imidazole ring, *Cg*2 that of the pyrimidine ring, *Cg*3 that of the phenyl ring. Dashed lines indicate stacking inter­actions. Symmetry codes: (i) 1 − *x*, −*y*, −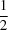 + *z*; (ii) 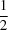 + *x*, 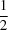 − *y*, *z*.

**Figure 4:**
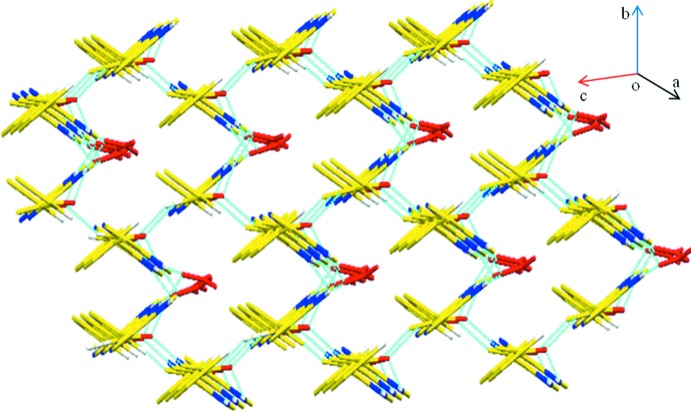
Overall packing in (I) viewed along the *a-*axis direction. Hydrogen bonds are drawn as light-blue dashed lines.

In the crystal structure of (I), the benzoyl­adeninium cations form base pairs *via* N—H⋯O and C—H⋯N hydrogen bonds (Table 1 ▸) involving the N1 and N6 atoms on the Watson–Crick face of the adenine ring system and the C16 and O1 atoms of the benzoyl ring of an adjacent benzoyl­adeninium cation. These result in the formation of a supra­molecular ribbon based on 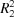
(9) rings, Fig. 2 ▸ *a*. The benzoyl­adeninum cations are also bridged by the O3 oxygen atoms of the nitrate anion, which acts as a bifurcated acceptor, forming N9—H9⋯O3 and N7—H7⋯O3 hydrogen bonds to generate a second ribbon motif, Fig. 2 ▸ *b*. π–π stacking inter­actions occur between the one face of the C11–C16 phenyl ring and the C4/C5/N7/C8/N9 imidazole ring with a relatively short centroid-to-centroid separation *Cg*1⋯*Cg*3^i^ = 3.4919 (17) Å [symmetry code: (i) 1 − *x*, −*y*, −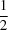 + *z*]. The other face of the phenyl ring makes offset π–π contacts with both the imidazole [*Cg*1⋯*Cg*3^ii^ = 3.7213 (17) Å] and the pyrimidine rings [*Cg*2⋯*Cg*3^ii^ = 3.5362 (16) Å; symmetry code (ii) 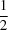 + *x*, 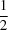 − *y*, *z*], Fig. 3 ▸. *Cg*1, *Cg*2 and *Cg*3 are the centroids of the imidazole, pyrimidine and phenyl rings, respectively. Similar contacts are found in related structures (Raghunathan & Pattabhi, 1981 ▸; Karthikeyan *et al.*, 2015 ▸). These various contacts combine to generate a three-dimensional supra­molecular architecture Fig. 4 ▸.

## Database Survey   

The crystal structures of a number of *N* ^6^-substituted adenines, adeninium salts and their metal complexes have been investigated in a variety of crystalline environments. Neutral mol­ecules include *N* ^6^-benzyl­adenine (Raghunathan *et al.*, 1983 ▸), *N* ^6^-furfuryladenine (Soriano-Garcia & Parthasarathy, 1977 ▸) and *N* ^6^-benzoyl­adenine (Raghunathan & Pattabhi, 1981 ▸). Recently our group reported the formation of two co-crystals, *N* ^6^-benzoyl­adenine–3-hy­droxy­pyridinium-2-carboxyl­ate (1:1) and *N* ^6^-benzoyl­adenine–dl-tartaric acid (1:1). In these, the benzoyl­adenine mol­ecule has a conformation similar to that reported for the neutral benzoyl­adenine crystal structure (Karthikeyan *et al.*, 2015 ▸). *N* ^6^-benzyl­adeninum salts with a wide variety of counter-anions have also been reported (Umadevi *et al.*, 2001 ▸; Xia *et al.*, 2010 ▸; Nirmalram *et al.*, 2011 ▸; Tamilselvi & Mu­thiah, 2011 ▸; McHugh & Erxleben, 2011 ▸; Stanley *et al.*, 2003 ▸). A variety of metal complexes of neutral *N* ^6^-benz­yl/furfuryladenines have been reported (Jennifer *et al.*, 2014 ▸), while structures of copper complexes of *N* ^6^-furfuryladeninium (Umadevi *et al.*, 2002 ▸) and *N* ^6^-benzyl­adeninium (Balasubramanian *et al.*, 1996 ▸) are also known.

## Synthesis and crystallization   

To a hot methanol solution of *N* ^6^-benzolyadenine (60 mg), a few drops of nitric acid were added. The resulting solution was warmed over a water bath for half an hour and then kept at room temperature for crystallization. After a week colourless prismatic crystals of (I) were obtained.

## Refinement   

Crystal data, data collection and structure refinement details are summarized in Table 2 ▸. Hydrogen atoms were readily located in difference Fourier maps and were subsequently treated as riding atoms in geometrically idealized positions, with C—H = 0.93 and N—H = 0.86 Å, and with *U*_iso_(H) = 1.2*U*_eq_(C, N).

## Supplementary Material

Crystal structure: contains datablock(s) I. DOI: 10.1107/S2056989015024871/sj5489sup1.cif

Structure factors: contains datablock(s) I. DOI: 10.1107/S2056989015024871/sj5489Isup2.hkl

Supporting information file. DOI: 10.1107/S2056989015024871/sj5489Isup3.pdf

Supporting information file. DOI: 10.1107/S2056989015024871/sj5489Isup4.cml

CCDC reference: 1444600

Additional supporting information:  crystallographic information; 3D view; checkCIF report

## Figures and Tables

**Table 1:** Hydrogen-bond geometry (Å, °)

*D*—H⋯*A*	*D*—H	H⋯*A*	*D*⋯*A*	*D*—H⋯*A*
N6—H6⋯O1^i^	0.86	2.33	3.135 (3)	156
N7—H7⋯O1	0.86	2.12	2.668 (3)	121
N7—H7⋯O3	0.86	1.99	2.709 (3)	140
N9—H9⋯O3^ii^	0.86	1.80	2.646 (3)	169
C16—H16⋯N1^iii^	0.93	2.55	3.426 (4)	157

Symmetry codes: (i) 
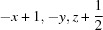
; (ii) 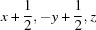; (iii) 
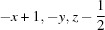
.

**Table 2:** Experimental details

Crystal data	
Chemical formula	C_12_H_10_N_5_O^+^·NO_3_^−^
*M*_r_	302.26
Crystal system, space group	Orthorhombic, *P* *n* *a*2_1_
Temperature (K)	293
*a*, *b*, *c* (Å)	12.7949 (10), 10.5639 (9), 9.6676 (6)
*V* (Å^3^)	1306.71 (17)
*Z*	4
Radiation type	Mo *K*α
μ (mm^−1^)	0.12
Crystal size (mm)	0.33 × 0.30 × 0.20

Data collection	
Diffractometer	Agilent SuperNova, Dual, Cu at zero, Atlas
Absorption correction	Multi-scan (*CrysAlis PRO*; Agilent, 2013 ▸)
*T*_min_, *T*_max_	0.791, 1.000
No. of measured, independent and observed [*I* > 2σ(*I*)] reflections	4891, 2559, 2080
*R*_int_	0.021
(sin θ/λ)_max_ (Å^−1^)	0.649

Refinement	
*R*[*F* ^2^ > 2σ(*F* ^2^)], *wR*(*F* ^2^), *S*	0.040, 0.097, 1.10
No. of reflections	2559
No. of parameters	200
No. of restraints	1
H-atom treatment	H-atom parameters constrained
Δρ_max_, Δρ_min_ (e Å^−3^)	0.19, −0.14

Computer programs: *CrysAlis PRO* (Agilent, 2013 ▸), *SIR97* (Altomare, 1999 ▸), *SHELXL2014*/7 (Sheldrick, 2015 ▸), *PLATON* (Spek, 2009 ▸) and *Mercury* (Macrae *et al.*, 2008 ▸).

## supplementary crystallographic information

### Crystal data

**Table:**

C_12_H_10_N_5_O^+^·NO_3_^−^	*D*_x_ = 1.536 Mg m^−^^3^
*M**_r_* = 302.26	Mo *K*α radiation, λ = 0.71073 Å
Orthorhombic, *P**n**a*2_1_	Cell parameters from 1553 reflections
*a* = 12.7949 (10) Å	θ = 3.7–27.6°
*b* = 10.5639 (9) Å	µ = 0.12 mm^−^^1^
*c* = 9.6676 (6) Å	*T* = 293 K
*V* = 1306.71 (17) Å^3^	Prism, colorless
*Z* = 4	0.33 × 0.30 × 0.20 mm
*F*(000) = 624	

### Data collection

**Table:**

Agilent SuperNova, Dual, Cu at zero, Atlas diffractometer	2080 reflections with *I* > 2σ(*I*)
Detector resolution: 10.4933 pixels mm^-1^	*R*_int_ = 0.021
ω scans	θ_max_ = 27.5°, θ_min_ = 2.9°
Absorption correction: multi-scan (*CrysAlis PRO*; Agilent, 2013)	*h* = −16→11
*T*_min_ = 0.791, *T*_max_ = 1.000	*k* = −13→9
4891 measured reflections	*l* = −12→12
2559 independent reflections	

### Refinement

**Table:**

Refinement on *F*^2^	Hydrogen site location: inferred from neighbouring sites
Least-squares matrix: full	H-atom parameters constrained
*R*[*F*^2^ > 2σ(*F*^2^)] = 0.040	*w* = 1/[σ^2^(*F*_o_^2^) + (0.0412*P*)^2^] where *P* = (*F*_o_^2^ + 2*F*_c_^2^)/3
*wR*(*F*^2^) = 0.097	(Δ/σ)_max_ < 0.001
*S* = 1.10	Δρ_max_ = 0.19 e Å^−^^3^
2559 reflections	Δρ_min_ = −0.14 e Å^−^^3^
200 parameters	Extinction correction: *SHELXL2014*/7 (Sheldrick, 2015), Fc^*^=kFc[1+0.001xFc^2^λ^3^/sin(2θ)]^-1/4^
1 restraint	Extinction coefficient: 0.0092 (16)

### Special details

**Table:**

Geometry. All e.s.d.'s (except the e.s.d. in the dihedral angle between two l.s. planes) are estimated using the full covariance matrix. The cell e.s.d.'s are taken into account individually in the estimation of e.s.d.'s in distances, angles and torsion angles; correlations between e.s.d.'s in cell parameters are only used when they are defined by crystal symmetry. An approximate (isotropic) treatment of cell e.s.d.'s is used for estimating e.s.d.'s involving l.s. planes.

### Fractional atomic coordinates and isotropic or equivalent isotropic displacement parameters (Å^2^)

**Table:**

	*x*	*y*	*z*	*U*_iso_*/*U*_eq_	
N1	0.7324 (2)	0.0197 (2)	0.8356 (3)	0.0543 (7)	
N3	0.8848 (2)	0.0766 (2)	0.7035 (3)	0.0546 (7)	
N6	0.56197 (18)	0.0580 (2)	0.7771 (2)	0.0458 (6)	
H6	0.5487	0.0479	0.8637	0.055*	
N7	0.67166 (18)	0.1685 (2)	0.5004 (2)	0.0437 (6)	
H7	0.6070	0.1798	0.4795	0.052*	
N9	0.84001 (19)	0.1733 (2)	0.4852 (2)	0.0467 (6)	
H9	0.9016	0.1874	0.4532	0.056*	
O1	0.48787 (16)	0.0648 (2)	0.5649 (2)	0.0540 (6)	
C2	0.8360 (3)	0.0298 (3)	0.8128 (4)	0.0591 (9)	
H2	0.8789	0.0000	0.8833	0.071*	
C4	0.8168 (2)	0.1171 (2)	0.6093 (3)	0.0425 (7)	
C5	0.7080 (2)	0.1128 (2)	0.6197 (3)	0.0379 (6)	
C6	0.6665 (2)	0.0629 (2)	0.7404 (3)	0.0420 (7)	
C8	0.7516 (2)	0.2016 (3)	0.4239 (3)	0.0478 (7)	
H8	0.7463	0.2400	0.3376	0.057*	
C10	0.4770 (2)	0.0675 (2)	0.6908 (3)	0.0405 (6)	
C11	0.3735 (2)	0.0836 (2)	0.7561 (3)	0.0406 (6)	
C12	0.3614 (2)	0.1411 (3)	0.8840 (3)	0.0471 (7)	
H12	0.4197	0.1645	0.9354	0.056*	
C13	0.2615 (3)	0.1635 (3)	0.9351 (3)	0.0559 (8)	
H13	0.2532	0.2035	1.0201	0.067*	
C14	0.1755 (3)	0.1272 (3)	0.8610 (4)	0.0580 (8)	
H14	0.1090	0.1420	0.8963	0.070*	
C15	0.1868 (3)	0.0689 (3)	0.7344 (4)	0.0581 (9)	
H15	0.1280	0.0437	0.6848	0.070*	
C16	0.2852 (2)	0.0480 (3)	0.6814 (3)	0.0483 (7)	
H16	0.2928	0.0100	0.5952	0.058*	
N10	0.5040 (2)	0.2616 (3)	0.2263 (3)	0.0563 (7)	
O2	0.5789 (2)	0.2331 (3)	0.1545 (3)	0.0904 (9)	
O3	0.51791 (16)	0.2830 (2)	0.3540 (2)	0.0660 (7)	
O4	0.4156 (2)	0.2712 (3)	0.1814 (3)	0.0993 (10)	

### Atomic displacement parameters (Å^2^)

**Table:**

	*U*^11^	*U*^22^	*U*^33^	*U*^12^	*U*^13^	*U*^23^
N1	0.0528 (16)	0.0628 (16)	0.0473 (14)	0.0062 (12)	−0.0050 (13)	0.0127 (14)
N3	0.0460 (14)	0.0608 (14)	0.0571 (17)	0.0070 (13)	−0.0043 (15)	0.0027 (15)
N6	0.0458 (13)	0.0589 (14)	0.0326 (12)	−0.0001 (11)	0.0032 (12)	0.0047 (11)
N7	0.0400 (13)	0.0536 (13)	0.0375 (13)	−0.0021 (11)	0.0002 (11)	0.0048 (11)
N9	0.0399 (13)	0.0549 (13)	0.0452 (14)	−0.0060 (11)	0.0022 (12)	−0.0012 (12)
O1	0.0503 (12)	0.0780 (15)	0.0338 (11)	−0.0144 (11)	0.0053 (10)	−0.0019 (10)
C2	0.055 (2)	0.068 (2)	0.054 (2)	0.0108 (16)	−0.0108 (17)	0.0123 (17)
C4	0.0442 (15)	0.0420 (13)	0.0415 (16)	0.0011 (13)	0.0011 (14)	−0.0050 (14)
C5	0.0403 (14)	0.0390 (12)	0.0344 (13)	0.0004 (12)	0.0004 (13)	−0.0039 (12)
C6	0.0448 (14)	0.0439 (14)	0.0375 (15)	0.0027 (12)	−0.0021 (15)	−0.0002 (13)
C8	0.0484 (17)	0.0536 (15)	0.0412 (16)	−0.0086 (14)	0.0019 (14)	0.0024 (14)
C10	0.0430 (15)	0.0427 (14)	0.0357 (15)	−0.0027 (12)	0.0024 (13)	0.0008 (13)
C11	0.0443 (15)	0.0428 (13)	0.0347 (13)	−0.0008 (12)	0.0049 (13)	0.0066 (12)
C12	0.0512 (16)	0.0514 (15)	0.0386 (15)	0.0021 (14)	0.0031 (15)	0.0021 (14)
C13	0.066 (2)	0.0601 (18)	0.0417 (17)	0.0105 (17)	0.0136 (16)	0.0033 (16)
C14	0.0522 (19)	0.0625 (18)	0.059 (2)	0.0077 (16)	0.0137 (18)	0.0131 (18)
C15	0.0493 (18)	0.0646 (18)	0.060 (2)	−0.0075 (16)	0.0022 (18)	0.0105 (18)
C16	0.0494 (18)	0.0532 (15)	0.0425 (15)	−0.0033 (14)	0.0018 (16)	0.0036 (15)
N10	0.0528 (17)	0.0612 (15)	0.0549 (16)	0.0087 (14)	0.0073 (15)	0.0001 (13)
O2	0.0774 (17)	0.1083 (19)	0.086 (2)	0.0132 (16)	0.0372 (16)	−0.0060 (17)
O3	0.0484 (13)	0.1011 (18)	0.0485 (13)	0.0120 (12)	−0.0007 (11)	0.0072 (14)
O4	0.0652 (16)	0.159 (3)	0.0738 (18)	0.0326 (19)	−0.0219 (15)	−0.0447 (19)

### Geometric parameters (Å, º)

**Table:**

N1—C6	1.330 (4)	C8—H8	0.9300
N1—C2	1.347 (4)	C10—C11	1.477 (4)
N3—C2	1.323 (4)	C11—C12	1.386 (4)
N3—C4	1.330 (4)	C11—C16	1.393 (4)
N6—C10	1.374 (4)	C12—C13	1.390 (4)
N6—C6	1.384 (4)	C12—H12	0.9300
N6—H6	0.8600	C13—C14	1.367 (5)
N7—C8	1.309 (3)	C13—H13	0.9300
N7—C5	1.376 (3)	C14—C15	1.378 (5)
N7—H7	0.8600	C14—H14	0.9300
N9—C8	1.312 (4)	C15—C16	1.378 (4)
N9—C4	1.371 (4)	C15—H15	0.9300
N9—H9	0.8600	C16—H16	0.9300
O1—C10	1.226 (3)	N10—O4	1.216 (3)
C2—H2	0.9300	N10—O2	1.222 (3)
C4—C5	1.397 (4)	N10—O3	1.268 (4)
C5—C6	1.386 (4)		
			
C6—N1—C2	118.9 (3)	N9—C8—H8	124.5
C2—N3—C4	111.0 (3)	O1—C10—N6	120.8 (3)
C10—N6—C6	127.3 (2)	O1—C10—C11	121.9 (3)
C10—N6—H6	116.4	N6—C10—C11	117.3 (2)
C6—N6—H6	116.4	C12—C11—C16	119.3 (3)
C8—N7—C5	108.9 (2)	C12—C11—C10	122.2 (3)
C8—N7—H7	125.6	C16—C11—C10	118.3 (3)
C5—N7—H7	125.6	C11—C12—C13	119.6 (3)
C8—N9—C4	107.9 (3)	C11—C12—H12	120.2
C8—N9—H9	126.0	C13—C12—H12	120.2
C4—N9—H9	126.0	C14—C13—C12	120.4 (3)
N3—C2—N1	128.6 (3)	C14—C13—H13	119.8
N3—C2—H2	115.7	C12—C13—H13	119.8
N1—C2—H2	115.7	C13—C14—C15	120.4 (3)
N3—C4—N9	126.7 (3)	C13—C14—H14	119.8
N3—C4—C5	126.3 (3)	C15—C14—H14	119.8
N9—C4—C5	107.0 (2)	C16—C15—C14	119.8 (3)
N7—C5—C6	137.6 (3)	C16—C15—H15	120.1
N7—C5—C4	105.2 (2)	C14—C15—H15	120.1
C6—C5—C4	117.1 (3)	C15—C16—C11	120.4 (3)
N1—C6—N6	115.0 (2)	C15—C16—H16	119.8
N1—C6—C5	118.0 (2)	C11—C16—H16	119.8
N6—C6—C5	126.9 (3)	O4—N10—O2	123.2 (3)
N7—C8—N9	110.9 (3)	O4—N10—O3	117.6 (3)
N7—C8—H8	124.5	O2—N10—O3	119.1 (3)
			
C4—N3—C2—N1	0.3 (5)	N7—C5—C6—N6	−1.3 (5)
C6—N1—C2—N3	−1.4 (5)	C4—C5—C6—N6	174.9 (3)
C2—N3—C4—N9	178.5 (3)	C5—N7—C8—N9	−0.9 (3)
C2—N3—C4—C5	−0.1 (4)	C4—N9—C8—N7	0.5 (3)
C8—N9—C4—N3	−178.7 (3)	C6—N6—C10—O1	9.9 (4)
C8—N9—C4—C5	0.1 (3)	C6—N6—C10—C11	−168.8 (2)
C8—N7—C5—C6	177.4 (3)	O1—C10—C11—C12	−150.8 (3)
C8—N7—C5—C4	1.0 (3)	N6—C10—C11—C12	27.9 (4)
N3—C4—C5—N7	178.2 (3)	O1—C10—C11—C16	24.4 (4)
N9—C4—C5—N7	−0.6 (3)	N6—C10—C11—C16	−156.9 (2)
N3—C4—C5—C6	0.9 (4)	C16—C11—C12—C13	−0.7 (4)
N9—C4—C5—C6	−178.0 (2)	C10—C11—C12—C13	174.5 (3)
C2—N1—C6—N6	−175.0 (3)	C11—C12—C13—C14	1.2 (4)
C2—N1—C6—C5	2.1 (4)	C12—C13—C14—C15	−0.5 (5)
C10—N6—C6—N1	−163.5 (2)	C13—C14—C15—C16	−0.6 (4)
C10—N6—C6—C5	19.7 (4)	C14—C15—C16—C11	1.1 (4)
N7—C5—C6—N1	−178.0 (3)	C12—C11—C16—C15	−0.4 (4)
C4—C5—C6—N1	−1.9 (4)	C10—C11—C16—C15	−175.8 (2)

### Hydrogen-bond geometry (Å, º)

**Table:**

*D*—H···*A*	*D*—H	H···*A*	*D*···*A*	*D*—H···*A*
N6—H6···O1^i^	0.86	2.33	3.135 (3)	156
N7—H7···O1	0.86	2.12	2.668 (3)	121
N7—H7···O3	0.86	1.99	2.709 (3)	140
N9—H9···O3^ii^	0.86	1.80	2.646 (3)	169
C16—H16···N1^iii^	0.93	2.55	3.426 (4)	157

Symmetry codes: (i) −*x*+1, −*y*, *z*+1/2; (ii) *x*+1/2, −*y*+1/2, *z*; (iii) −*x*+1, −*y*, *z*−1/2.
