# Supplementary material for: Supra­molecular hydrogen-bonding patterns in the N(9)—H protonated and N(7)—H tautomeric form of an N6-benzoyl­adenine salt: N 6-benzoyl­adeninium nitrate
Source: Acta Crystallogr E Crystallogr Commun. 2016 Jan 9;72(Pt 2):140–3. doi: 10.1107/S2056989015024871 (PMC4770981; doi:10.1107/S2056989015024871)
Supplement: Supplementary file 3 [file e-72-00140-Isup3.pdf]

**Table.3.** Conformation of *N*$^{6}$-benzoyladeninum molecule in the salt of N6-benzoyladeninum nitrate (NA)(I)

| Crystals | Dihedral angle (°) | | | | Torsion angle(°) |
|---|---|---|---|---|---|
| | Primidine ring /imdazole ring of adenine (N1-N3-C2-C4-C5-C6/N7-N9-C4-C5-C8) | Purine ring /benzene ring (N1-C2-N3-C4-C5-C6-N7-C8-N9/C11-C12-C13-C14-C15-C16) | Purine ring /amide (N1-C2-N3-C4-C5-C6-N7-C8-N9/N6-H6-C10-O1) | Benzene ring /amide (C11-C12-C13-C14-C15-C16/N6-H6-C10-O1) | (C6-N6-C10-C11) |
| $N^{6}$-benzoyladenine–3-hydroxypyridinium-2-carboxylate(1:1) ( Ref. a) | 3.00(9) | 0.94(8) | 21.20(17) | 21.45(18) | -176.24(16) |
| $N^{6}$-benzoyladenine–DL-tartaric acid(1:1)( Ref. a) | 2.26(10) | 9.77(8) | 2.93(18) | 11.35(9) | -179.08(17) |
| $N^{6}$-benzoyladeninium Nitrate (present) | 1.34(14) | 52.25(12) | 23.7(2) | 29.2(2) | -168.8(2) |

Ref: a) Karthikeyan *et al.*, 2015.

**Table.4.** Comparison of dihedral angles (°) between the adenine plane and phenyl ring plane in some related structures

| **Name of the Compound** | **Molecule A (°)** | **Molecule B (°)** | **Reference** |
|---|---|---|---|
| N6-benzyladenine | 78.5(3) | | a |
| N6-benzyladeninium oxalate | 49.03(8) | | b |
| N6-benzyladeninium p-toluenesulfonate | 82.76(11) | | c |
| N6-benzyladeninium sulphosalicylate monohydrate | 77.04(10) | | d |
| N6-benzyladeninium 3-hydroxypicolinate | 67.91(12) | 68.27(13) | e |
| N6-benzyladeninium nitrate | 76.64(16) | | f |
| N6-benzyoladenine | 7.4 | | g |
| N6-benzyoladenine 3-hydroxypyridinium-2-carboxylate | 1.54(7) | | h |
| N6-benzyoladenine (DL)-tartaric acid | 9.74(7) | | i |
| N6-benzoyladeninium nitrate | 51.57(10) | | Present |

Ref: a) Raghunathan *et al.*, 1983; b) McHugh & Erxleben,2011; c) Tamilselvi & Muthiah, 2011; d) Xia *et al.*, 2010; e &f) Nirmalram *et al.*, 2011; i) Raghunathan & Pattabhi, 1981; h &i) Karthikeyan *et al.*, 2015.
